# *TP53* Splice Mutations Have Tumour-Independent Effects on Genomic Stability and Prognosis: An In Silico Study

**DOI:** 10.3390/ijms262412080

**Published:** 2025-12-16

**Authors:** Apeksha Arun Bhandarkar, Noah Ethan Kelly-Foleni, Debina Sarkar, Aaron Jeffs, Tania Slatter, Antony Braithwaite, Sunali Mehta

**Affiliations:** 1 Department of Pathology, University of Otago, Dunedin 9016, New Zealand; bhaap437@student.otago.ac.nz (A.A.B.); kelno418@student.otago.ac.nz (N.E.K.-F.); debina.sarkar@otago.ac.nz (D.S.); aaron.jeffs@otago.ac.nz (A.J.); antony.braithwaite@otago.ac.nz (A.B.); 2 Maurice Wilkins Centre for Biodiscovery, University of Auckland, Auckland 1142, New Zealand; 3 Medical Laboratory Science, University of Otago, Dunedin 9016, New Zealand; tania.slatter@otago.ac.nz

**Keywords:** p53, mutation, splice, isoforms

## Abstract

The tumour suppressor *TP53* (tumor protein p53) is a master regulator of cell cycle, DNA repair, and apoptosis, and its mutation is a hallmark of cancer, with individual mutations exerting distinct effects on tumour biology. Despite accounting for ~7% of all *TP53* variants, splice site mutations remain the least studied class, and their functional and clinical consequences are poorly understood. We analyzed 25,058 *TP53* variants (18,562 somatic; 6496 germline) to characterize the frequency, molecular impact, transcriptional effects, genomic instability, and clinical outcomes of splice mutations. These alterations showed distinct distributions and substitution patterns between germline and somatic contexts and were frequently associated with copy number alterations, reduced *TP53* mRNA, and variable protein expression. Transcriptomic profiling identified two transcriptional phenotypes: one with global suppression of canonical p53 target genes and another with mixed activation and repression independent of tumour type. Genomic instability was elevated in a subset of splice-mutant tumours, correlating with increased relapse risk, while other splice mutations showed lower instability but divergent clinical outcomes, including unexpectedly poor prognoses. Our findings fill a critical knowledge gap, defining the biological and clinical spectrum of *TP53* splice site mutations and highlighting their potential as prognostic biomarkers and therapeutic targets in precision oncology.

## 1. Introduction

Splicing defects play a pivotal role in cancer by disrupting the expression of key genes associated with the ‘hallmarks of cancer’ [1,2,3]. These defects arise from mutations in splicing factors (e.g., SF3B1 and U2AF1) or cis-regulatory elements, resulting in dysfunctional proteins, oncogenic isoforms, or nonfunctional transcripts [reviewed in [3]]. Canonical splice site mutations at GT–AG dinucleotides further exacerbate aberrant splicing, leading to exon skipping, intron retention, or the activation of cryptic splice sites. In tumour suppressor genes such as *TP53*, these alterations may profoundly reshape transcript and protein output, ultimately influencing cell fate.

**Figure 1:**
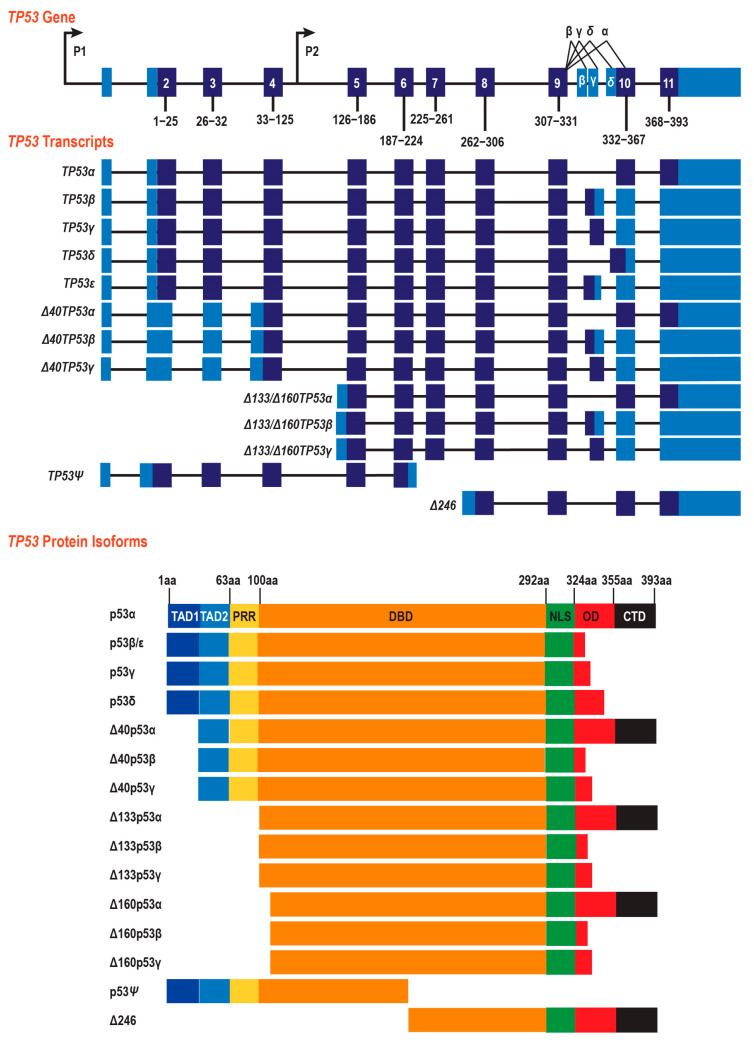
Schematic representing the structure of the *TP53* gene, derived mRNA transcripts, and known protein isoforms. Top Panel: *TP53* gene (light blue—5’UTR and 3’UTR; dark blue—coding exons). P1 and P2 indicate promoters 1 and 2 of *TP53*. Middle Panel: *TP53* transcripts encoding *FLTP53α/β/γ/δ/ε*, Δ*40TP53α/β/γ*, Δ*133/*Δ*160TP53α/β/γ, p53-psi*, and *delta246* protein isoforms (light blue—5’UTR and 3’UTR; dark blue—coding exons). Bottom Panel: *TP53* isoforms encoding *FLTP53α/β/γ/δ/ε*, Δ*40TP53α/β/γ*, Δ*133/*Δ*160TP53α/β/γ, p53-ψ*, and Δ*246* protein isoforms showing protein domains (TAD1 (dark blue) and TAD2 (light blue)—transactivation domain; PRR (yellow)—proline-rich region; DBD (orange)—DNA binding domain; NLS (green)—nuclear localisation signal; OD (red)—oligomerisation domain; and CTD (black)—C-terminal domain).

The *TP53* gene encodes p53, a central regulator of genomic integrity, apoptosis, and senescence [4,5]. Its locus is structurally complex, with two promoters and extensive alternative splicing, and these together generate at least 12 protein isoforms (Figure 1). Promoter 1, upstream of exon 1, produces full-length p53 (p53α) and N-terminally truncated Δ40p53 isoforms, while an internal promoter in intron 4 gives rise to Δ133p53 and Δ160p53 transcripts [6]. Additional C-terminal diversity arises from alternative 3′ splicing between exon 9 and exon 10, producing p53α/β/γ variants. Isoforms lacking N-terminal domains (Δ40p53, Δ133p53, and Δ160p53) or bearing C-terminal truncations (p53β and p53γ) differ in terms of transcriptional activity, oligomerization, and interaction with full-length p53, allowing the fine-tuning of stress responses but also creating vulnerability to splice disruption [7]. Notably, Δ133p53 suppresses apoptosis and senescence to promote proliferation and invasion, whereas p53β enhances senescence [7,8,9]. In addition, Δ40p53, which lacks part of the transactivation domain, can act as a dominant-negative inhibitor of full-length p53, reducing the cell’s ability to respond to genotoxic stress [10]. Splice mutations that alter this balance can therefore shift cells toward oncogenesis.

Large-scale cancer genomics studies estimate that ~7% of *TP53* mutations occur at canonical splice sites or adjacent splice regions [11]. Several pan-cancer analyses have highlighted the enrichment of synonymous mutations at critical splice junctions, including the 5′ end of exon 6 and the 3′ ends of exons 4 and 9 [12,13,14]. These alterations can abolish functional p53 activity, act in a dominant-negative manner against p53α, or generate novel gain-of-function isoforms [12,13,14]. Notably, aberrant splicing can produce transcriptionally inactive variants such as p53Ψ, which promote pro-metastatic behaviour [15]. Far from being rare events, splice-altering *TP53* mutations are increasingly recognized across tumour types and carry distinct clinical implications.

Multiple tumour-specific studies underscore the biological and prognostic impact of *TP53* splice mutations. In colorectal cancer (CRC), the unbiased sequencing of coding and splice regions identified splice mutations in 4% of cases, mutually exclusive from exon mutations. These generated aberrant transcripts, often truncated or oncogenic, yet converged on transcriptional effects similar to exonic mutations. Strikingly, in stage II CRC, splice mutations were associated with worse relapse-free survival compared to both wild-type and exon-mutated tumours [16]. In pediatric adrenocortical tumours (ACT), germline variants at the exon 4–intron 4 junction were enriched and resulted in unstable proteins with loss of function, but without classic Li-Fraumeni syndrome phenotypes, suggesting alternative mechanisms of cancer susceptibility [17].

Comparable findings have emerged in other cancers. In diffuse astrocytomas, splice site mutations accounted for a notable fraction of *TP53* alterations (three of eight cases with mutations), emphasizing that they are more frequent than previously reported [18]. In ovarian cancer, novel splice variants such as p53δ were associated with an impaired response to platinum-based chemotherapy and significantly worse recurrence-free and overall survival [19]. Similarly, alternative splicing events in exon 9β and 9γ have been linked to unbalanced isoform expression and potentially deleterious effects, with both somatic and germline variants clustering in this hotspot region [20]. Clinical reports further illustrate the complexity of splice mutations: for example, a donor site mutation in intron 6 (c.672 + 1G>A) produced an in-frame insertion with low mutant transcript expression but tumour-specific loss of the wild-type allele [21]. Likewise, a de novo synonymous germline mutation (c.672G>A) was identified in a 17 year old with two primary sarcomas, creating a 5′ cryptic splice site bound by U1 that shifted splicing by 5 bp. This frameshift truncated the protein at residue 246, disrupting the DNA-binding domain of p53 [22]. Adding further complexity, translation from an alternative initiation site at codon 246 was recently shown to generate a novel isoform, Δ246p53, which is conserved across vertebrates, induced by DNA damage, and capable of driving senescence and suppressing tumour growth through modulation of p21 activity [23]. Together, these findings emphasize the diversity of molecular outcomes and clinical consequences arising from *TP53* splice mutations.

More recently, deep sequencing of over 55,000 tumours identified recurrent intronic substitutions at position c.375+5G in intron 4 of *TP53*, affecting 0.2% of *TP53*-mutated cancers across multiple sites. These variants were strongly associated with the loss of heterozygosity or a second hit, produced aberrant transcripts with loss of normal isoforms, and consistently resulted in absent p53 protein expression by immunohistochemistry [24]. In head and neck squamous cell carcinoma cell lines, splice mutations generated truncated proteins undetectable by standard immunohistochemistry, highlighting how reliance on exon sequencing or IHC underestimates their prevalence [25]. Importantly, this work highlighted a clinically relevant diagnostic pitfall: tumours with p53 loss by IHC but no detectable exonic mutation may harbour deleterious intronic splice variants, reinforcing the importance of sequencing beyond coding regions.

Despite growing recognition, the systematic characterization of *TP53* splice mutations remains limited. Unlike missense variants, which are well studied, comprehensive splicing assays have only recently been applied to clinically relevant variants. Minigene-based studies show that most predicted splice-altering variants generate aberrant transcripts consistent with loss of function, underscoring the need to integrate splicing data into variant classification frameworks [24]. Collectively, splice mutations in *TP53* are more common, biologically consequential, and clinically relevant than previously appreciated. They contribute to tumorigenesis through the loss of tumour suppressor function, dominant-negative effects, or oncogenic splice products, and their presence correlates with worse outcomes across cancers. Here, we present the first comprehensive data-mining analysis of *TP53* splice site mutations across germline and somatic contexts, demonstrating that donor and acceptor mutations have distinct molecular and clinical consequences. Our findings highlight the underappreciated role of *TP53* splicing alterations in cancer biology and their potential relevance for prognosis and therapy.

## 2. Results

### 2.1. Frequency and Characteristics of Reported TP53 Splice Mutations in Somatic and Germline Tumours

**Figure 2:**
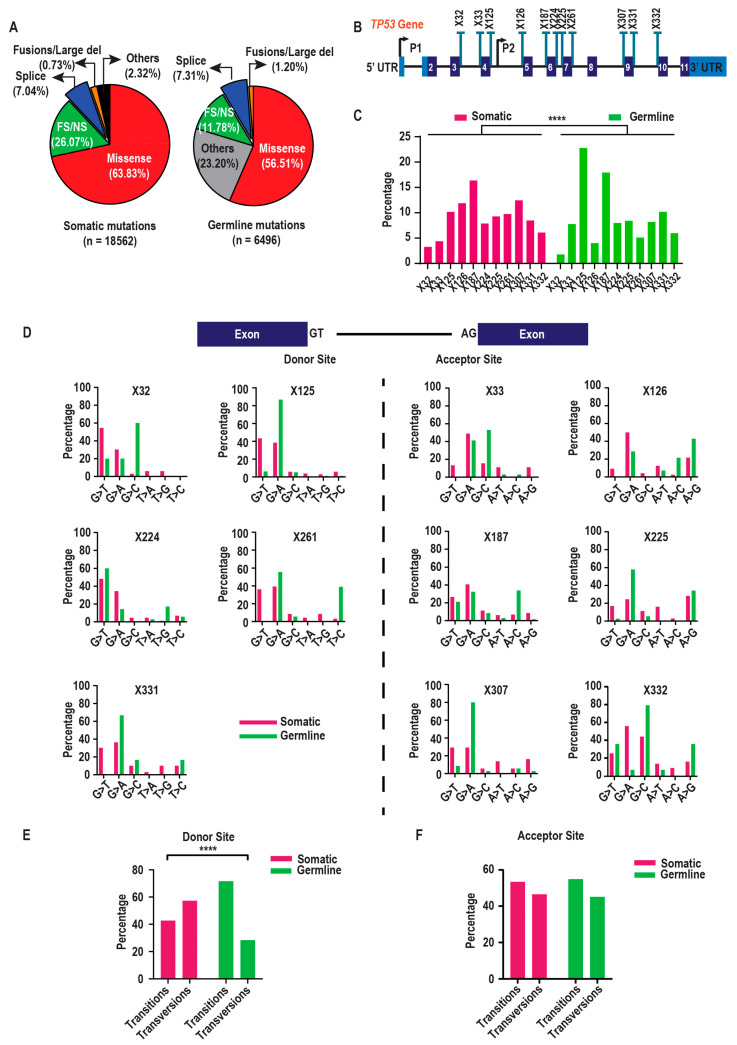
*TP53* splice mutations and their characteristics from somatic and germline databases. (**A**) The pie chart represents the distribution of mutation types (missense and frameshift/nonsense (FS/NS), splice, fusions/large del, in-frame deletion and in-frame insertion, translation start site, nonstop mutations, 5’UTR and 3’UTR, synonymous and intronic variants) reported in somatic (TCGA Pan-Atlas Cancer Dataset, the MSK-MetTropism Metastatic Dataset, and the Tumour Mutation Burden and Immunotherapy Dataset) and germline (IARC database and gnomAD database) contexts. (**B**) Schematic representing the structure of the *TP53* gene with location of splice mutations (blue bars). (**C**) Bar chart showing the percentage distribution of individual *TP53* splice mutations across somatic (TCGA Pan-Atlas Cancer Dataset, the MSK-MetTropism Metastatic Dataset, and Tumour Mutation Burden (TMB) and Immunotherapy Dataset) and germline (IARC database and gnomAD database) contexts. Significance was determined using the chi-square test and *p* < 0.0001 (****) is considered significant. Donor site mutations include X32 (somatic: *n* = 40, germline: *n* = 8), X125 (somatic: *n* = 125; germline: *n* = 103), X224 (somatic: *n* = 97; germline: *n* = 36), X261 (somatic: *n* = 120; germline: *n* = 23), and X331 (somatic: *n* = 104; germline: *n* = 46), while acceptor site mutations include X33 (somatic: *n* = 54; germline: *n* = 35), X126 (somatic: *n* = 146; germline: *n* = 18), X187 (somatic: *n* = 201; germline: *n* = 18), X225 (somatic: *n* = 114; germline: *n* = 38), X307 (somatic: *n* = 153; germline: *n* = 37), and X332 (somatic: *n* = 75; germline: *n* = 27). (**D**) Schematic showing bases that constitute donor and acceptor splice sites flanking exons. Bar graphs depicting the percentage of base substitution types—G>A/T/C and T>A/G/C at donor sites, and A>T/C/G and G>T/A/C at acceptor sites—for individual *TP53* splice site mutations. (**E**,**F**) Bar graphs showing the percentage of base substitutions resulting in transitions or transversions in (**E**) donor sites and (**F**) acceptor sites. Significance was determined using (Fisher’s exact test) and **** *p* <0.001—significant.

Analysis of large-scale cancer germline and somatic datasets revealed that approximately 7% of reported *TP53* mutations occur at canonical splice sites or splice regions (Figure 2A; Supplementary Table S1). These mutations are particularly significant given the complexity of the *TP53* locus, which encodes multiple transcripts (Figure 1).

Reported *TP53* splice site mutations are located at both donor and acceptor sites flanking several introns: introns 3 (X32–X33), 4 (X125–X126), 6 (X224–X225), and 9 (X331–X332); acceptor sites flanking introns 5 (X187) and 8 (X307); and the donor site flanking intron 7 (X261) (Figure 2B). These mutations occur in both somatic and germline tumours, though their frequencies vary across datasets (Figure 2C). A significant difference in mutation frequency between somatic and germline datasets was observed (Chi-square test, *p* < 0.0001). Specifically, mutations at X33, X125, X187 and X331 are more common in germline tumours, whereas X32, X126, X261, and X307 are more prevalent in somatic tumours. Mutations at X224, X225, and X332 occur at similar frequencies in both datasets (Figure 2C).

At canonical splice sites, donor sequences are typically GT/CA, while acceptor sequences generally consist of AG/TC [26,27] (Figure 2D, top panel). At donor sites, nucleotide substitutions such as G>A/T/C and T>A/G/C are common, while acceptor sites frequently harbour A>T/C/G and G>T/A/C mutations, with variation between germline and somatic datasets. Notably, the nucleotide immediately adjacent to the exon (“G”) is more frequently mutated than the second nucleotide (“T”), which accounts for less than 10% of donor site mutations. Substitution patterns also differ by site and dataset. For instance, G>A substitutions at X125, X261, and X331 are more prevalent in germline tumours, whereas the same change at X32 and X224 occurs more frequently in somatic tumours. Similarly, G>T mutations at X32, X125, and X224 are observed in both datasets, while the same change at X261 and X331 occurs only in somatic tumours. Exceptionally high G>C substitutions at X32 and T>C substitutions at X261 are enriched in the germline dataset compared to the somatic dataset (Figure 2D, donor sites).

Acceptor sites show similar trends, with the nucleotide closest to the exon (“G”) more frequently mutated than the second position (“A”) (Figure 2D, acceptor sites). Exceptions include A>G mutations at X126 and X225, and an A>C mutation at X187. The A>G change at X126 is more prevalent in germline tumours, whereas this trend is not observed for X225. Likewise, the A>C mutation at X187 is enriched in germline tumours (Figure 2D, acceptor sites). Differences in G>T, G>A, and G>C substitutions are also evident. G>T mutations at X33 and X126 are observed exclusively in somatic tumours, while G>T mutations at other acceptor sites are present in both datasets. G>A is the most frequently reported substitution at most acceptor sites in both datasets, except for X332, where G>C predominates in germline tumours (Figure 2D, acceptor sites).

Beyond site-specific patterns, broader mutational trends are apparent. At donor sites, transversions are more common than transitions in somatic datasets. However, the bias is stronger towards transitions being more common than transversions in germline datasets (Chi-square test, *p* < 0.001; Figure 2E), suggesting site-specific mutational preferences. This trend is not observed at acceptor sites (Figure 2F).

Overall, these findings highlight distinct mutational patterns at *TP53* splice sites, revealing positional and nucleotide-specific biases as well as notable differences between somatic and germline tumours.

### 2.2. Association of TP53 Splice Mutations with Copy Number, mRNA Expression, and Protein Levels

We evaluated the association of individual *TP53* splice mutations with p53 mRNA expression, protein levels, and copy number alterations (deletion or gain/amplification) compared to tumours with no *TP53* mutations, using data from the pan-cancer TCGA dataset. Overall, splice mutations showed significant associations with all molecular readouts, though the direction and magnitude varied (Table 1).

*TP53* splice mutations were associated with frequent copy number alterations, reduced p53 mRNA levels (odds ratio [OR] 0.46), and a weaker reduction in protein levels (OR 0.74). Except for X32, all splice mutations showed elevated odds of copy number deletion (OR 1.48–8.56), significant for X33, X126, and X261 (*p* < 0.001). Copy number (CN) gain was significantly enriched at X187, X224, X261, and X331 (OR 1.77–10.43).

At the site level, X125 and X225 did not exhibit significant CN alterations but showed reductions in terms of both mRNA and protein expression (ORs 0.34–0.40, *p* < 0.001). X331 showed CN gain and reduced mRNA (OR 0.34, *p* < 0.001) with a marginal protein decrease (OR 0.57, *p* = 0.05). X187, X224, and X307 were associated with CN gain, significantly reduced mRNA (ORs 0.34–0.38, *p* < 0.001), and non-significant protein decreases (ORs 0.57–0.78). X332 also showed mRNA loss (OR 0.38, *p* < 0.001) but a non-significant protein increase (OR 2.37).

In contrast, X126 and X261 demonstrated CN alterations with modest, non-significant increases in both mRNA (ORs 1.34–1.63) and protein (ORs 2.09–2.10). X32 showed neutral CN status with reduced mRNA (OR 0.88) but elevated protein (OR 2.57), while X33 displayed CN deletion, increased mRNA (OR 1.61), and slightly reduced protein (OR 0.73).

In summary, *TP53* splice mutations were closely linked to altered gene dosage and reduced mRNA expression, yet their impact on protein abundance was more variable and splice site specific. Mutations such as X125 and X225 showed concordant reductions in transcript and protein levels, consistent with a loss-of-function effect, despite there being no CN changes. In contrast, X32 and X33 exhibited striking discordance between mRNA and protein abundance. This pattern may reflect splice-dependent post-transcriptional regulation, including reduced translation efficiency, the production of unstable p53 isoforms, or enhanced protein turnover via ubiquitin-mediated degradation, all of which can uncouple protein levels from both gene dosage and mRNA abundance [28]. These mechanisms highlight that *TP53* splice mutations influence tumour biology through multiple layers of regulation, including gene dosage, transcript stability, translational control, and protein degradation, underscoring the complexity of the splicing-driven disruption of p53 function.

### 2.3. Differential Impact of TP53 Splice Mutations on p53 Signalling

**Figure 3:**
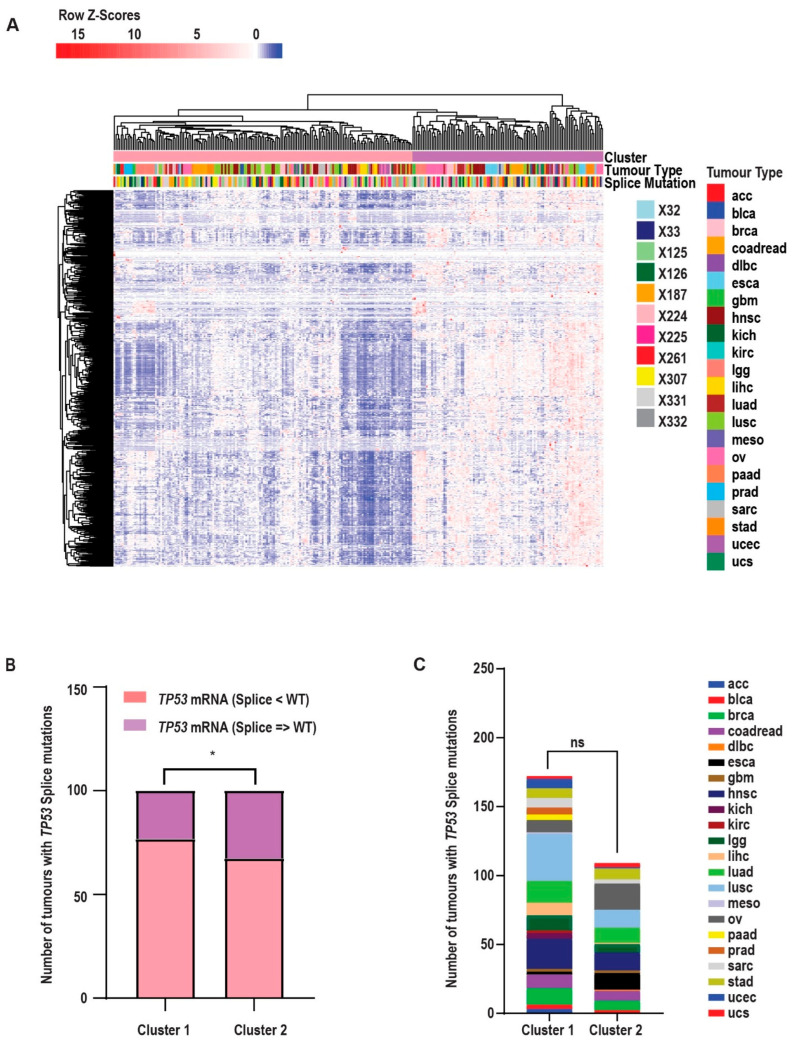
*TP53* splice mutations result in aberrant p53 signalling. (**A**) Pan-cancer heatmap showing the mean-centred mRNA expression of genes directly regulated by or associated with p53. Rows represent genes; columns represent individual tumour samples. Both rows and columns were hierarchically clustered using Euclidean distance and complete linkage. Column sidebars indicate the following: two expression clusters identified by k-means clustering (Cluster 1: widespread downregulation of p53 signalling genes; Cluster 2: selective downregulation with concurrent upregulation of others), tumour type, and the presence of *TP53* splice mutations. (**B**) Distribution of tumours with *TP53* splice mutations based on p53 mRNA levels compared to tumours with wild-type *TP53*. Mutations in X125, X187, X224, X225, X307, and X331 are associated with decreased p53 mRNA expression, while mutations in X32, X33, X126, X261, and X332 show expression levels equal to or greater than those in the wild type. Fisher’s exact test was used to assess statistical significance (* *p* = 0.035; *p* < 0.05 considered significant). (**C**) Number of tumours with *TP53* splice mutations by tumour type in Cluster 1 and Cluster 2. The chi-square test for trend was used; *p* = 0.7294 (ns = not significant).

To investigate the impact of individual *TP53* splice site mutations on p53 signalling, we analyzed the mRNA expression profiles of 663 genes known to be direct transcriptional targets of *TP53* or associated with its regulatory network. This analysis was conducted using the hierarchical clustering of tumour samples harbouring *TP53* splice mutations. The results revealed that tumours could be broadly categorized into two distinct clusters based on the expression patterns of p53 signalling-related genes (Figure 3A).

Tumours in Cluster 1 exhibited a global downregulation of genes associated with p53 signalling and a higher proportion of splice mutations for which overall *TP53* mRNA expression was reduced compared to wild-type (WT) *TP53* tumours. In contrast, tumours in Cluster 2 displayed a more heterogeneous expression pattern, characterized by the selective downregulation of certain p53 target genes alongside the upregulation of others. This cluster also included a higher proportion of splice mutations associated with *TP53* mRNA expression levels that were comparable to or greater than those observed in WT-*TP53* tumours (Figure 3A,B). Notably, these clustering patterns and their associated expression profiles were not attributable to the tumour anatomical site, indicating that the observed differences in splice mutation frequency and transcriptional impact were not driven by the cancer type (Figure 3A,C). These results underscore the transcriptional heterogeneity among *TP53* splice mutations and highlight their potential to differentially disrupt p53 signalling, independent of tumour type.

### 2.4. TP53 Splice Mutations Confer Heterogeneous Effects on Tumour Biology and Disease Progression

**Figure 4:**
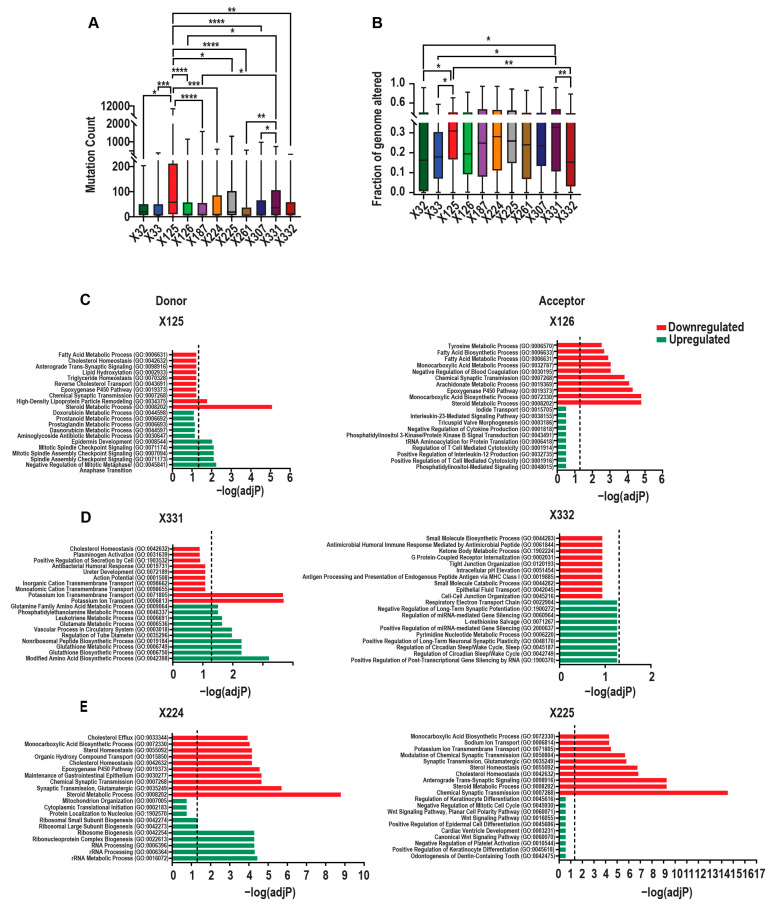
Genomic instability and biological pathways associated with *TP53* splice mutations. (**A**,**B**). Box plots showing the distribution of (**A**) mutation count and (**B**) FGA, for tumours with individual *TP53* splice mutations including X32 (*n* = 25), X33 (*n* = 30), X125 (*n* = 88), X126 (*n* = 100), X187 (*n* = 134), X224 (*n* = 62), X225 (*n* = 71), X261 (*n* = 78), X307 (*n* = 87), X331 (*n* = 58), and X332 (*n* = 49). (**A**,**B**). Significance was determined using Kruskal–Wallis test, *p* < 0.05 was considered significant. * *p* < 0.05, ** *p* < 0.01, *** *p* < 0.001 and **** *p* < 0.0001. Datasets used TCGA pan-cancer dataset and MSK-MetTropism datasets from cBioPortal. (**C**) X125 and X126, (**D**) X331 and X332, (**E**) X224 and X225. EnrichR: GO Biological pathways associated with top significantly associated (padj ≤ 0.01) genes with donor (X125, X224, X331) and acceptor site (X126, X225, and X332) splice mutations compared to tumours with wild-type *TP53*. Downregulated—red (LFC ≤ −0.5, padj < 0.01). Upregulated—green (LFC ≥ 0.5, padj < 0.01).

To further characterize the molecular features of tumours harbouring *TP53* splice mutations, we investigated whether individual splice variants were associated with genomic alterations indicative of genomic instability. Specifically, we analyzed the tumour mutation burden (TMB) and the fraction of genome altered (FGA) using data from the pan-cancer TCGA and MSK cohorts. Tumours with X125 and X331 splice mutations exhibited the highest mutation burdens and FGA scores, significantly exceeding most other splice variants (Figure 4A,B), consistent with pronounced genomic instability. In contrast, X126 and X332 tumours showed lower mutation counts, with X332 displaying significantly reduced FGA and X126, showing modestly lower FGA relative to X125 and X331 (Figure 4A,B). Tumours with X187, X224, X225, and X307 mutations also exhibited FGA scores comparable to those of X125 and X331, suggesting that these variants may similarly contribute to elevated genomic instability.

To gain insight into the pathways underlying these differences and their clinical implications, we focused on *TP53* splice mutations forming donor–acceptor pairs: X125/X126, X224/X225, and X331/X332, since other sites lack corresponding counterparts. Differential gene expression analysis was performed by comparing tumours carrying these variants to *TP53* wild-type tumours, identifying pathways enriched among significantly up- or downregulated genes (LFC ≥ 0.5 or ≤−0.5, padj < 0.05). To determine which aspects of p53 activity were lost, retained, or potentially novel in tumours harbouring specific *TP53* splice mutations, we performed Spearman’s correlation analysis between *TP53* mRNA expression and genome-wide gene expression (ρ ≥ 0.5 or ≤−0.5, *p* < 0.01). Significantly correlated genes were subjected to pathway enrichment analysis, and the resulting pathways were overlaid with a curated list of pathways enriched using the 663 known *TP53* target genes and regulatory network members. This approach allowed us to distinguish canonical *TP53*-regulated processes that were preserved, disrupted, or altered in a mutation-specific manner. Additionally, the clinical significance of these splice variants was evaluated using disease-free survival (DFS) data from cBioPortal, where events were defined as recurrence, progression, or death.

**Figure 5:**
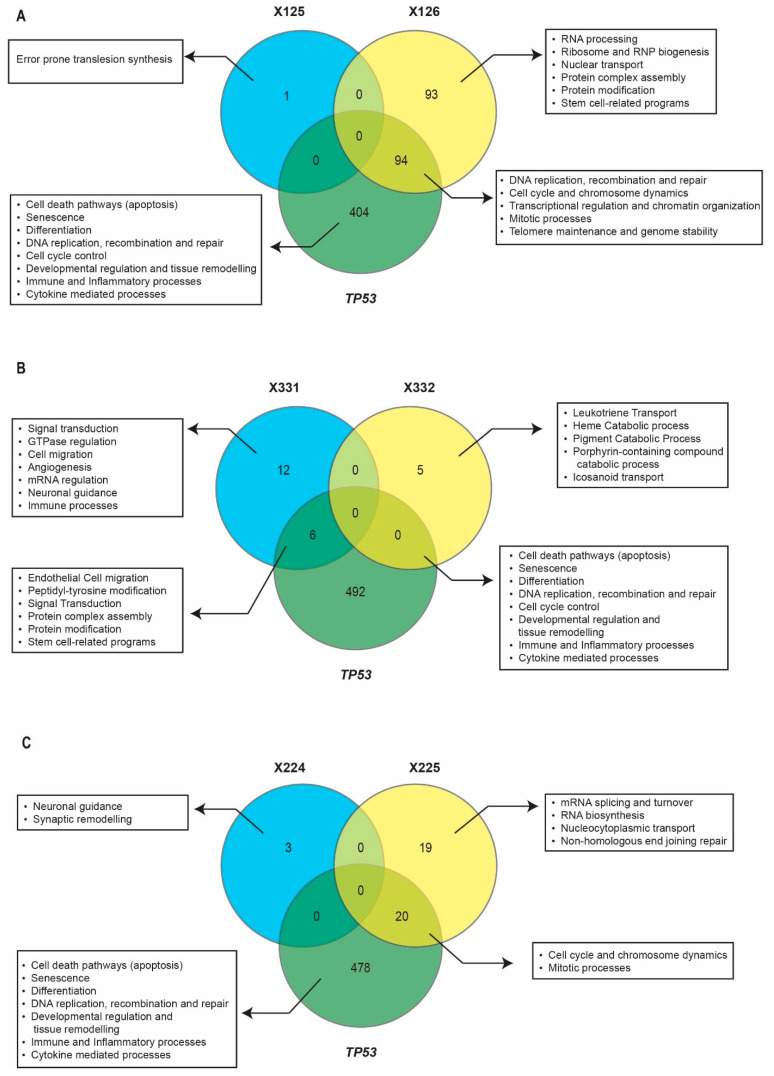
Canonical *TP53* pathways and unique biological processes associated with *TP53* splice mutations. (**A**–**C**) Venn diagram showing unique and overlapping GO Biological Process (*p* ≤ 0.05) for acceptor and donor pairs with GO Biological Processes enriched for *TP53* target genes and regulatory network members. (**A**) X125–X126, (**B**) X331-X332 and (**C**) X224 and X225. Venny [29] was used to generate the Venn diagrams.

Consistent with their high TMB and FGA, X125 tumours exhibited upregulation of mitotic spindle checkpoint signalling and downregulation of steroid metabolism compared to *TP53* wild-type tumours (Figure 4C). Moreover, *TP53* mRNA within X125 tumours uniquely correlated with error-prone translesion synthesis while lacking associations with canonical apoptosis, cell cycle control, or DNA replication, recombination and repair pathways (Figure 5A). Clinically, patients with X125 (OR = 3.16, *p* < 0.05) were significantly associated increased DFS events (Table 2). By contrast, consistent with their low TMB and FGA, X126 tumours showed only a downregulation of steroid metabolism compared to tumours with wild-type *TP53* (Figure 4C). Interestingly, *TP53* mRNA from the X126 tumours correlated with genes involved in RNA processing and splicing, transcriptional regulation and chromatin organization, ribosome and RNP biogenesis, nuclear transport, cell cycle and chromosome dynamics, mitotic processes, telomere maintenance and genome stability, DNA replication, recombination and repair, protein complex assembly and modification, and stem-cell-related programmes, while lacking associations with canonical apoptotic, cell cycle regulatory, and differentiation pathways (Figure 5A). Moreover, X126 tumours were associated with increased DFS events (OR = 3.50, *p* < 0.005, Table 2). These findings suggest that the adverse clinical behaviour of X126 tumours may be driven not by classical genomic instability mechanisms, but by alternative regulatory programmes.

Like X125, *TP53* mRNA in X331 tumours lacked association with pathways involved in canonical apoptosis, cell cycle control, or DNA replication, recombination and repair pathways (Figure 5B), supporting its role in the accumulation of higher TMB and FGA. Furthermore, *TP53* mRNA in X331 tumours correlated with pathways related to signal transduction, GTPase regulation, cell migration, angiogenesis, mRNA regulation, neuronal guidance, and immune processes (Figure 5B). In addition, X331 tumours showed an upregulation of modified amino acid biosynthesis as well as glutamate and leukotriene metabolism, alongside a downregulation of potassium ion transport compared to *TP53* wild-type tumours (Figure 4D). Clinically, patients with X331 (OR = 3.10, *p* < 0.05) tumours were significantly more likely to experience recurrence, progression, or death (Table 2). In contrast, X332 tumours also exhibited low TMB and FGA but showed no significant enrichment of biological processes compared to *TP53* wild-type tumours (Figure 4D). Moreover, *TP53* mRNA in X332 tumours was associated with leukotriene transport, heme and porphyrin catabolism, and icosanoid transport pathways, but lacked an association with canonical apoptosis, cell cycle control, or DNA replication, recombination and repair pathways (Figure 5B). Despite this, clinically, X332 tumours had similar DFS compared to wild-type *TP53* tumours (Table 2). Taken together, these observations indicate that the X332 *TP53* variant does not display clear functional or clinical effects and may resemble a biologically mild alteration within this tumour context.

Tumours with X224 and X225 mutations exhibited intermediate FGA values and the downregulation of steroid metabolism and cholesterol homeostasis compared to tumours with wild-type *TP53* (Figure 5C). Tumours with X224 *TP53* mutations additionally showed the enrichment of RNA processing and biogenesis among upregulated genes (Figure 4E). Furthermore, *TP53* mRNA in X224 lacked correlation with canonical apoptosis, cell cycle control, or DNA replication, recombination and repair pathways, and was uniquely associated with pathways related to neuronal guidance and synaptic remodelling (Figure 5C). On the other hand, *TP53* mRNA in X225 tumours mainly lacked a correlation with apoptosis but retained its association with the cell cycle and was also associated with pathways involved in mRNA splicing and turnover, RNA biosynthesis, nucleocytoplasmic transport, and nonhomologous end-joining DNA repair (Figure 5C). Despite the presence of intermediate genomic instability and alterations in transcriptional programmes, neither X224 nor X225 were significantly associated with poor DFS (Table 2). Overall, these findings suggest that X224 and X225 may introduce modest transcriptional and genomic changes; however, these alterations do not appear to translate into clinically significant increases in recurrence, progression, or death compared to tumours with wild-type *TP53*.

Taken together, these findings demonstrate that distinct *TP53* splice mutations drive unique molecular programmes that not only influence genomic instability but also differentially impact patient outcomes, underscoring the biological and clinical heterogeneity conferred by *TP53* splicing events.

## 3. Discussion

Splice site mutations are emerging as critical modulators of cancer biology, reshaping gene expression, isoform balance, and downstream cellular programmes. Our study demonstrates that *TP53* splice mutations, which account for ~7% of all *TP53* alterations in both somatic and germline datasets, are highly heterogeneous in both molecular and clinical consequences. While our data support the distinct effects of donor and acceptor disruptions, these observations remain correlative, and the underlying mechanisms require further experimental validation. Importantly donor and acceptor site mutations at canonical splice junctions are not functionally equivalent, and their effects span transcriptional output, genomic instability, and patient outcomes. These patterns are broadly consistent with established differences in spliceosome interactions at 5′ donor versus 3′ acceptor sites, although the extent to which these mechanisms operate specifically at *TP53* remains to be empirically determined. Donor disruptions typically impair exon definition and reduce full-length protein production, whereas acceptor disruptions frequently redirect splicing toward alternative acceptor sites, preserving exon inclusion but altering isoform composition [30,31]. Future studies will be required to directly test whether these mechanistic models explain the divergent phenotypes observed across donor–acceptor mutation pairs in *TP53*.

Beyond site-specific effects, broader mutational trends are apparent. At donor sites, somatic mutations more often involve transversions, whereas germline donor sites are biased toward transitions. These patterns may reflect underlying biological mechanisms or selective pressures during tumour development. However, these differences remain speculative, and integrating environmental, genomic, and epigenomic data will be needed to determine whether specific mutational processes preferentially target donor sites.

Disruption of the donor site, as exemplified by the X125 splice mutation, was associated with reduced p53 transcript and protein levels, increased genomic instability, and poor patient outcomes, consistent with a loss-of-function mechanism of the full-length p53 protein [4,5]. This interpretation is further supported by the upregulation of mitotic spindle checkpoint signalling [32] and the downregulation of steroid metabolic pathways [33] in X125 tumours relative to wild-type *TP53* tumours. Moreover, *TP53* mRNA in X125 tumours was enriched for error-prone translesion synthesis and lacked association with canonical p53 programmes involved in DNA replication, recombination, repair, and genome maintenance, aligning with the elevated TMB and FGA observed in these tumours. A similar pattern was seen with the donor site mutation X331, which also showed reduced p53 transcript and protein abundance, heightened genomic instability, and poor clinical outcomes, further supporting a loss-of-function phenotype [4,5]. A testable hypothesis arising from these observations is that donor site mutations reduce full-length p53 dosage to a threshold that compromises genome maintenance; isogenic systems carrying engineered donor site variants could directly measure this.

In contrast, the disruption of the acceptor site by the X126 splice mutations exhibited increased mRNA and protein abundance [1,2,6], and reduced genomic instability, but were still associated with poor DFS, suggesting a gain of function effect. This phenotype was supported by the absence of genomic instability pathways in X126 tumours and the enrichment of *TP53* mRNA in canonical pathways regulating DNA replication, recombination, repair, mitosis, and genome stability, as well as RNA-splicing processes. Notably, truncated isoforms such as Δ40p53, Δ133p53, and Δ166p53 have been implicated in facilitating DNA repair, promoting cell survival, and inhibiting apoptosis following DNA damage [7,8,9,10,34], but the specific isoforms produced in X126 tumours and their functional relevance remain unvalidated. This supports a hypothesis that X126 exerts its effects through altered isoform ratios rather than genomic instability, an idea that can be directly tested through long-read RNA sequencing and isoform-specific RNA/protein quantification in isogenic systems carrying engineered acceptor site variants.

Mutations at the adjacent donor and acceptor sites X224–X225 also resulted in reduced p53 transcript and protein expression, consistent with loss of full-length p53 function [4,5]. Although X224–X225 tumours exhibited moderate levels of FGA compared to X125 and X331, they did not significantly influence recurrence or progression. Likewise, the acceptor site mutation X332 did not substantially alter mRNA or protein levels, displayed low genomic instability, and had no significant impact on clinical outcomes, though sample size was limited. However, given the limitations of our data, we cannot yet determine the precise isoform signatures generated by each variant, nor can we definitively link specific phenotypes to changes in the isoform balance.

Overall, these data highlight that the contrasting phenotypes of *TP53* splice variants arise from position-dependent alterations in p53 dosage and isoform architecture, reinforcing the need for splice-aware variant interpretation in clinical genomics. However, given the limitations of our data, we cannot yet determine the precise isoform signatures generated by each variant, nor can we definitively link specific phenotypes to changes in isoform balance.

From a translational standpoint, multiple splice mutations are associated with significantly poorer DFS than wild-type *TP53* tumours. However, exon-focused sequencing has limited sensitivity for detecting these alterations: while splice variants located within 1 bp of the exon–intron junction can be called with high confidence, detection rapidly declines as the mutation lies further into the intron [35]. However, 84% of splice mutations reported in our analyzed dataset lie beyond this window and may be missed. As a result, conventional exon-targeted sequencing and immunohistochemistry may substantially underestimate both the prevalence and clinical impact of splice mutations as has been previously suggested by Eicheler et al. [25]. Incorporating splice-aware sequencing and isoform-level analyses will therefore be essential to improving diagnostic precision. A testable clinical hypothesis is that expanded sequencing approaches will reveal additional splice site variants associated with adverse outcomes.

Beyond detection, *TP53* splice site mutations may also guide therapeutic strategies: donor site variants such as X125 and X331 appear vulnerable to approaches exploiting genomic instability, whereas the acceptor site mutation X126, despite its low genomic instability, may require interventions that target altered p53 dosage and isoform imbalance. These possibilities require experimental validation, including drug response testing in isogenic models engineered to carry specific splice site mutations. These patterns highlight that *TP53* splice mutations reshape tumour biology through both quantitative and qualitative changes in p53 isoform architecture.

A central limitation of our study is the inability of short-read sequencing to determine which *TP53* isoforms are produced, or in what ratios, given the complexity of the locus [6]; thus, the precise isoform signatures associated with individual splice site mutations remain unresolved. Addressing this gap will require long-read RNA sequencing, and isoform-specific RNA [36] and protein quantification [5] to define the full-length and truncated isoform repertoire generated by each mutation. Isogenic cellular models carrying specific splice site alterations will then be essential for establishing causality between splice disruption, altered isoform architecture, and downstream phenotypes. Functional assays of p53 transcriptional activity, DNA damage response, cell fate, and genome stability will further clarify whether the effects of splice site mutations arise from changes in p53 dosage rather than classical loss-of-function mechanisms. Complementary transcriptional and chromatin-mapping approaches will help delineate how splice-site-specific differences in isoform architecture reshape *TP53*-driven signalling networks, while analyses of protein stability, localization, and post-translational regulation will identify additional mechanisms modulating p53 dosage. Finally, in vivo validation using patient-derived organoids or xenografts, together with isoform ratio correlations in clinical cohorts, will be necessary to establish the clinical significance of splice-dependent alterations in p53 isoform architecture.

Together, these results generate clear, testable hypotheses regarding how individual splice site mutations reshape p53 dosage and isoform balance, but the mechanistic basis remains to be definitively established. Donor site mutations such as X125 and X331 are associated with reduced p53 dosage and genomic instability, whereas the acceptor site mutation X126 is linked instead to altered isoform balance despite preserved genome maintenance. These interpretations remain provisional and require direct isoform-resolved validation. As precision oncology advances, incorporating splice-aware variant interpretation and elucidating the biological consequences of splice site disruption will be critical for improving risk stratification and informing targeted therapeutic strategies in p53-driven cancers.

## 4. Methods and Materials

### 4.1. Retrieval of Somatic TP53 Mutations Reported in the cBioPortal Database

The integrative analysis of reported somatic *TP53* mutations was performed on the pan-cancer database from The Cancer Genome Atlas (TCGA) [37], the Memorial Sloan Kettering (MSK)–MetTropism Metastatic Dataset [38], and the Tumour Mutation Burden and Immunotherapy Dataset (TMBID) [39] using cBioportal [40,41,42] (accessed and downloaded in September, 2024). This database facilitates large-scale statistical analysis and graphical viewing of tumour changes at mRNA and protein level across the following tumour types: Adrenocortical Carcinoma (acc), Bladder Urothelial Carcinoma (blca), Breast Invasive Carcinoma (brca), Cervical Squamous Cell Carcinoma (cesc), Cholangiocarcinoma (chol), Colorectal Adenocarcinoma, Diffuse Large B-Cell Lymphoma (dlbc), Esophageal Adenocarcinoma (esca), Glioblastoma Multiforme (gbm), Head and Neck Squamous Cell Carcinoma (hnsc), Kidney Chromophobe (kich), Kidney Renal Clear Cell Carcinoma (kirc), Kidney Renal Papillary Cell Carcinoma (kirp), Acute Myeloid Leukemia (laml), Liver Hepatocellular Carcinoma (lihc), Brain Lower Grade Glioma (lgg), Lung Adenocarcinoma (luad), Lung Squamous Cell Carcinoma (lusc), Mesothelioma (meso), Ovarian Serous Cystadenocarcinoma (ov), Pancreatic Adenocarcinoma (paad), Pheochromocytoma and Paraganglioma (pcpg), Prostate Adenocarcinoma (prad), Sarcoma (sarc), Skin Cutaneous Melanoma (skcm), Stomach Adenocarcinoma (stad), Testicular Germ Cell tumours (tgct), Thyroid Carcinoma (thca), Thymoma (thym), Uterine Corpus Endometrial Carcinoma (ucec), and Uterine Carcinosarcoma (ucs). For our analysis, the data from the three somatic datasets (TCGA, MSK-MetTropism, and TMBID) from cBioPortal were combined under somatic category. A total of 11,378 samples were derived from the TCGA dataset, out of which 7128 were genetically wild type (WT) and 4250 carried a *TP53* gene mutation (~37%). Similarly, a total of 26,796 samples were derived from the MSK-MetTropism dataset, of which 13,325 were genetically WT and 13,471 had a *TP53* gene mutation (~49%). Lastly, from the TMBID, 1764 sample details were derived, with 923 as genetically WT and 841 having *TP53* gene mutations (~48%).

### 4.2. Retrieval of Germline TP53 Mutations Reported in the International Agency for Research on Cancer (IARC) *TP53* Database

The reported germline *TP53* mutations were downloaded from IARC database [43] (accessed and downloaded in October 2024) along with their characteristics and family history. The IARC database is a publicly available database for the analysis and interpretation of the biological and clinical impacts of *TP53* mutations across various cancer types, including cancer of the adrenal gland, bladder, bones, brain, breast, lung, cervix, uterus, colorectum, stomach, esophagus, head and neck, kidney, lymph nodes, ovary, pancreas, prostate gland, skin, thyroid, and ureter. A total of 4455 samples were retrieved from the database under germline variants. Additionally, 2041 germline *TP53* mutations were also downloaded from gnomAD database [44] (accessed and downloaded in July 2025), which is a publicly available resource for the analysis and interpretation of human genetic variation, mainly focusing on population-level allele frequencies and not associating variants with specific cancer types. For the two germline datasets, the retrieved data comprised individuals that had *TP53* mutations and were combined under the germline category.

### 4.3. TP53 Mutation Frequency

We categorized the *TP53* mutations from somatic and germline datasets as missense, frameshift/nonsense, splice, fusions/large deletions, and other mutations (in-frame, translation start site, nonsense/frameshift mutations, 5’UTR and 3’UTR, synonymous, and intronic variants). The most frequently occurring mutations in *TP53* were considered as hotspot mutations, namely R175, G245, R248, R273, R282, and Y220 [45] and are included as part of the missense mutations. The splice mutations comprised X32 (*n* = 40 somatic; *n* = 8 germline), X33 (*n* = 54 somatic; *n* = 35 germline), X125 (*n* = 125 somatic; *n* = 103 germline), X126 (*n* = 146 somatic; *n* = 18 germline), X187 (*n* = 201 somatic; *n* = 81 germline), X224 (*n* = 97 somatic; n = 36 germline), X225 (*n* = 114 somatic; *n* = 38 germline), X261 (*n* = 120 somatic; *n* = 23 germline), X307 (*n* = 153 somatic; *n* = 37 germline), X331 (*n* = 104 somatic; *n* = 46 germline), and X332 (*n* = 75 somatic; *n* = 27 germline). These sites represent commonly observed splice mutations in each dataset.

To assess the frequency and distribution of *TP53* splice site mutations across multiple tumour types, we analyzed the types of genetic alterations at each splice site position in both the somatic and germline datasets. These included single nucleotide polymorphisms (SNPs), dinucleotide polymorphisms (DNPs), deletions (del), and insertions (ins). However, for the splice site analysis, only SNPs were included. These were further categorized as transversions (G>T, G>C, T>A, T>G) and transitions (G>A, T>C) to compare mutation patterns between somatic and germline datasets. Statistical comparisons were performed using the chi-square test to determine whether there were significant differences in nucleotide changes between the somatic and germline mutations, as well as between the donor and acceptor splice sites.

### 4.4. Molecular Characteristics of TP53 Splice Mutations

The molecular characteristics analyzed included mutation count and fraction of the genome altered (FGA), which were available only in the somatic datasets, TCGA and MSK-MetTropism. The mutation count refers to the total number of mutations present in a tumour sample, while FGA represents the percentage of the genome affected by changes such as copy number variations, structural rearrangements, insertions, deletions, and single nucleotide variants. Together, these metrics provide an overall measure of genomic instability.

Study IDs and sample IDs for each *TP53* mutation category, including splice site mutations, were retrieved from the raw data and used for statistical analysis of mutation count and genomic alterations. Data from the TCGA and MSK-MetTropism datasets were included, focusing only on primary tumour samples and excluding metastatic samples. The Kruskal–Wallis test was applied to assess whether significant differences (*p* < 0.05) existed between the different *TP53* mutation groups.

Copy number, mRNA expression, and protein levels were assessed in primary tumour samples using data from TCGA. Logistic regression was performed to evaluate the association between the presence of individual splice mutations and *TP53* CNV, mRNA expression, or protein levels. Models were fitted using the glm() function in R (version 4.4.3 [46]) with a binomial family for the outcome.

To investigate the impact of *TP53* splice site mutations on p53 signalling, a reference list of 663 genes known to be *TP53* targets or associated with *TP53* expression was compiled (Supplementary Table S2). This list was curated using multiple resources available through the EnrichR database [47,48,49], including the ARCHS4 RNA-seq gene–gene co-expression matrix [50], EnrichR gene–gene co-occurrence matrix [47,48,49], Tagger literature gene–gene co-mentions matrix [51], and GeneRIF literature gene–gene co-mentions matrix [52]. mRNA expression data for these genes were extracted from the TCGA Pan-Cancer dataset for tumour samples harbouring *TP53* splice site mutations. Expression values were log-transformed and mean-centred to normalize them across the samples. Hierarchical clustering (Euclidean distance and complete linkage) was performed to group samples based on similarity in gene expression profiles. Clustering was applied to both rows (genes) and columns (samples), allowing the identification of distinct expression patterns and potential subgroups among *TP53* splice mutant tumours. Heatmaps were generated to visualize co-expression patterns and to explore the association between *TP53* splice site mutations and transcriptional dysregulation of p53-associated genes. R (version 4.4.3 [46]) was used to normalize the data, perform hierarchical clustering, and generate a heatmap. The chi-square test was employed to assess the significance of splice mutation frequency, while the chi-square test for trends was used to evaluate the distribution of splice mutations by tumour type across the two clusters identified in the heatmap. Statistical analyses and graphical representations were conducted using GraphPad Prism (version 10.4.2), R (version 4.4.3 [46]), and Adobe Illustrator (version 29.5.1 (64 bit)).

### 4.5. Differential Gene Expression, Correlation Analysis, and Pathway Enrichment Analysis

RNA-sequencing data for the pan-cancer TCGA cohort were downloaded from the GDC data portal (downloaded in March 2025). Differential gene expression analysis was performed using DESeq2, comparing tumours harbouring *TP53* splice mutations (X125, X126, X224, X225, X331, and X332) to tumours with wild-type *TP53*. Genes with log2 fold change (LFC) ≥ 0.5 and adjusted *p*-value (padj) < 0.01 were considered significantly upregulated, while genes with LFC ≤ –0.5 and padj < 0.01 were considered significantly downregulated and were used to identify pathways differentially expressed in individual splice mutations compared to tumours with wild-type *TP53*. Pathway enrichment analysis was conducted using EnrichR database [47,48,49], with GO Biological Process considered significant at padj < 0.05.

Spearman’s correlation analysis was performed between *TP53* gene expression and all other genes across the genome using RNA-sequencing data from the pan-cancer TCGA cohort, focusing on tumours harbouring *TP53* splice mutations (X125, X126, X224, X225, X331, and X332). Genes with a correlation coefficient (ρ) ≥ 0.5 or ≤−0.5 and *p*-value < 0.01 were considered positively or negatively correlated with *TP53* mRNA expression for each individual splice site mutation. These correlated genes were then subjected to pathway enrichment analysis using the EnrichR database [47,48,49], with GO Biological Process considered significant at *p* < 0.001.

### 4.6. Clinical Outcome Analysis

Clinical data were obtained from cBioPortal for matched tumour types carrying individual *TP53* splice mutations and wild-type *TP53* (WT-*TP53*) tumours. For each group, the proportion of patients who experienced recurrence, progression, or death was determined. Differences in event frequencies between splice mutation groups and WT-*TP53* tumours were assessed using chi-square tests, and odds ratios (ORs) were calculated with the Baptista–Pike method in GraphPad Prism (Version 10.6.1 (892)). A *p*-value < 0.05 was considered statistically significant, with * *p* < 0.05 and ** *p* < 0.005 indicating levels of significance.

## Figures and Tables

**Table 1:** Association of individual *TP53* splice mutations with copy number alterations, mRNA expression, and protein abundance. Odds ratios (OR) with 95% confidence intervals (CI) and logistic regression *p*-values are shown for each *TP53* splice mutation for copy number deletion, copy number gain/amplification (Gain/AMP), mRNA expression, and protein expression. ORs were derived from logistic regression models, with values < 1 indicating decreased odds and > 1 indicating increased odds relative to tumours with no *TP53* mutations. Significance codes: *** *p* < 0.0001, ** *p* < 0.001, * *p* < 0.01, “.” *p* < 0.1 and >0.05, and ns > 0.1 not significant.

*TP53* Splice Mutations	Copy Number Deletion			Copy Number Gain/AMP			mRNA			Protein		
	OR	95% CI	Significance	OR	95% CI	Significance	OR	95% CI	Significance	OR	95% CI	Significance
X32 (*n =* 9)	0.62	0.03−0.62	ns	0.00	0−8.63 × 10^3^	ns	0.88	0.36−0.88	ns	2.57	0.46−2.57	ns
X33 (*n* = 15)	7.08	2.23−7.08	**	0.00	0−8.63 × 10^3^	ns	1.61	0.86−1.61	ns	0.73	0.31−0.73	ns
X125 (*n* = 54)	1.48	0.67−1.48	ns	2.58	0.57−2.58	ns	0.36	0.27−0.36	***	0.40	0.25−0.40	***
X126 (*n* = 35)	8.56	3.23−8.56	***	2.92	0.41−2.91	ns	1.63	1−1.63	.	2.10	0.93−2.10	.
X187 (*n* = 38)	1.73	0.64−1.73	ns	9.44	3.10−9.44	***	0.34	0.25−0.34	***	0.57	0.30−0.57	ns
X224 (*n* = 25)	2.24	0.52−2.24	ns	6.61	0.84−6.51	*	0.38	0.24−0.38	***	0.69	0.27−0.69	ns
X225 (*n* = 27)	1.99	0.70−1.99	ns	1.77	0.09−1.88	ns	0.34	0.24−0.34	***	0.37	0.22−0.37	***
X261 (*n* = 20)	7.71	2.20−7.71	**	9.82	2.13−9.82	**	1.34	0.77−1.34	ns	2.09	0.79−2.09	ns
X307 (*n* = 27)	2.05	0.63−2.05	ns	1.92	0.10−1.92	ns	0.38	0.25−0.38	***	0.78	0.33−0.78	ns
X331 (*n* = 27)	2.21	0.65−2.21	ns	10.43	2.38−10.43	**	0.34	0.23−0.34	***	0.49	0.25−0.49	.
X332 (*n* = 20)	2.66	0.69−2.66	ns	4.06	0.56−4.06	ns	0.53	0.33−0.53	*	2.37	0.77−2.37	ns

**Table 2:** Association of individual *TP53* splice mutations with disease recurrence/progression/death. Odds ratios (OR) with 95% confidence intervals (CI) and chi-square test *p*-values are shown for X125, X126, X224, X225, X331, and X332 *TP53* splice mutation for disease relapse. ORs were derived from the Baptista–Pike method, with values < 1 indicating decreased odds and >1 indicating increased odds relative to tumours with no *TP53* mutations. Significance codes: ** *p* < 0.01, * *p* < 0.05, and ns > 0.1 not significant.

*TP53* Splice Mutations	Likelihood of Relapse		
	OR	95% CI	Significance
X125	3.16	0.96–10.51	*
X126	3.50	1.4–8.7	**
X224	1.16	0.19–7.09	ns
X225	1.27	0.37–4.6	ns
X331	3.10	1.05–9.1	*
X332	0.72	0.12–4.41	ns

## Data Availability

Data presented in the study is available in publicly accessible repositories and the original data are openly available in cBioPortal [https://www.cbioportal.org/] (accessed on 1 September 2024) the GDC data portal [https://portal.gdc.cancer.gov/] (accessed on 1 March 2025), the IARC database [https://tp53.cancer.gov/] (accessed on 1 October 2024), and the gnomAD database [https://gnomad.broadinstitute.org/] (accessed on 1 July 2025).

## Supplementary Materials

The following supporting information can be downloaded at: https://www.mdpi.com/article/10.3390/ijms262412080/s1.
