# Supplementary material for: TP53 Splice Mutations Have Tumour-Independent Effects on Genomic Stability and Prognosis: An In Silico Study
Source: Int J Mol Sci. 2025 Dec 16;26(24):12080. doi: 10.3390/ijms262412080 (PMC12732612; doi:10.3390/ijms262412080)
Supplement: Supplementary file 1 [file ijms-26-12080-s001.zip › Supplementary Tables.pdf]

Table S1. List of reported TP53 mutations occurring at canonical splice sites or splice regions from both somatic cBioPortal - The Cancer Genome Atlas (TCGA), Memorial Sloan-Kettering (MSK)-MetTropism Metastatic Dataset and Tumour Mutation Burden and Immunotherapy Dataset (TMBID) and germline - IARC and gnomAD Databases.

Splice Site	HGVS Nomenclature	Somatic Frequency	Germline Frequency	Variant Type
X10	c.-29+1G>A, c.-28-352_177del, c.-28-2_-25del, c.-28-3C>G, c.-28-4G>A	2	3	SNP, DEL
X14	c.40_75-11del	1	-	DEL
X23	c.68_74+1dup	1	-	DUP
X24	c.70_74+26del	1	-	DEL
X25	c.75-1G>T, c.75-2A>G, c.75-1G>C, c.75-1G>A, c.75-19_78del, c.75-5_81del, c.75-10_81del	10	1	SNP, DEL
X26	c.77_96+8del	1	-	DEL
X27	c.81_253del	1	-	DEL
X32	c.96G>A, c.96+1G>T, c.96+1G>A, c.96+1G>C, c.96+3A>T, c.96+2T>G, c.96+1_96+6del, c.96+96_1dup, c.96_96+1delinsAA, c.96_96+1delinsAT, c.96_96+1delinsTT, c.74+2_96+29del	40	8	SNP, DEL, INS, DUP
X33	c.97-1G>A, c.97-1G>C, c.97-1G>T, c.97-2A>G, c.97-2A>T, c.97-11C>G, c.97-2del, c.97-8_106del, c.97-4_131del, c.97-6_129del, c.97-12_97-2del, c.97-2_97-1insC, c.97-50_111del, c.97-16_134del, c.97-1_102delinsTTCCCA, c.97-3C>T, c.97-4A>T, c.97-5T>G, c.97-6C>G, c.97-6C>T, c.97-8A>G	54	35	SNP, DEL, INS
X71	c.213_375+43del	1	-	DEL
X110	c.329_375+27del	1	-	DEL
X112	c.336_375+127del	1	-	DEL
X113	c.339_375+130del	1	-	DEL
X123	c.367_376+5del, c.368_375+130del	2	-	DEL
X124	c.371_375+10delinsTCA	1	-	DEL

X125	c.375G>T, c.375G>A, c.375G>C, c.375+1G>T, c.375+1G>A, c.375+1G>C, c.375+8G>A, c.375+8G>T, c.375+1del, c.375+2T>A, c.375+2T>C, c.375+2T>G, c.375+1_375+18del, c.375_375+1delinsTT, c.374+375+12del, c.375_375+1delinsAA, c.375+335_560-4del, c.375+267_470del, c.375_375+3del, c.375+1_375+2insN, c.374_375+7del	125	103	SNP, DEL, INS
X126	c.376-1G>T, c.376-1G>A, c.376-1G>C, c.376-2A>C, c.376-2A>G, c.376-2A>T, c.376-1_391del, c.376-15_382del, c.375-8_389del, c.376-285_478del, c.376-4_379del, c.376-6_383del, c.376-2_387del, c.376-11_379del, c.376-2_380delinsTCT, c.376-56_456del, c.376-1del, c.376-73_502del, c.376-9_390del, c.376-8_380del, c.376-10_384del, c.376-56_604del, c.376-32_376-2del, c.376-14_410del, c.376-17_376delinsTCTGC, c.376-12_376-1del, c.376-1_376delinsAA, c.376-2_378del, c.376-8_384del, c.376-2_376-1insN, c.376-1_377del, c.376-12_378del, c.376-2dup, c.376-4A>C, c.376-4A>T, c.376-7C>T	146	18	SNP, DEL, INS, DUP
X146	c.437_560-26del	1	-	DEL
X153	c.457_672+44del	1	-	DEL
X162	c.486_600del	1	-	DEL
X169	c.505_560-24del, c.375+191_507del	2	-	DEL
X178	c.532_559+6del	1	-	DEL
X181	c.541_672+19del	1	-	DEL
X183	c.547_559+6del, c.547_559+33delinsA	2	-	DEL
X186	c.557_559+11del	1	-	DEL
X187	c.559G>C, c.559+1G>T, c.559+1G>A, c.559+1G>C, c.559+2T>G, c.559+2T>C, c.559+2T>A, c.560-1G>A, c.560-1G>C, c.560-1G>T, c.560-2A>C, c.559+2del, c.560-2A>G , c.560-2A>T, c.560-2A>C, c.560-3T>G, c.560-17_567del, c.560-21_565del, c.560-7_564del, c.560-1_560delinsAA, c.560-8_565del, c.560-10_597del, c.559+33_585delinsAA, c.560-4_574del, c.560-14_560-2del, c.560-4_571del, c.559+2dup, c.559+35_569del, c.560-29_587del, c.560-23_573del, c.560-15_585del, c.559+2del, c.559_559+1delinsTA, c.560-5_560-1del, c.560-11_560-1del, c.559+7A>T, c.559+8G>T, c.560-11_560-8del, c.560-5C>T, c.560-6T>C, c.560-8G>C, c.559+1_559+ 2insCGGAGGTTGTGAGGCGCTGCCCCCACCATGAGCGCTGCTCAGATAACGATGG,	201	81	SNP, DEL, INS, DUP
X194	c.581_672+261del	1	-	DEL

X196	c.587_672+194del	1	-	DEL
X206	c.616_672+2del	1	-	DEL
X219	c.657_782+160del	1	-	DEL
X221	c.661_672+12del	1	-	DEL
X223	c.667_672+1dup	1	-	AMP
X224	c.672G>T, c.672G>A, c.672+1G>A, c.672+1G>T, c.672+1G>C, c.672+2T>A, c.672+2T>G, c.672+2T>C, c.672+1del, c.672_672+38del, c.672+1_672+79del, c.671_672+206del, c.672+46_829del, c.672+8_672+22del c,672+8T>G	97	36	SNP, DEL
X225	c.673-1G>A, c.673-1G>C, c.673-1G>T, c.673-2A>G, c.673-2A>C, c.673-2A>T, c.673-5_682del, c.673-1_673delinsTT, c.673-10_675del, c.673-188_775del, c.673-8_677del, c.673-2_677del, c.673-3T>G, c.673-3T>C, c.673-4C>G, c.673-5C>A, c.673-5C>G	114	38	SNP, DEL, INS
X237	c.709_782+90del	1	-	DEL
X243	c.728_782+4del	1	-	DEL
X246	c.738_782+87del	1	-	DEL
X255	c.764_787del	1	-	DEL
X257	c.769_782+4del	1	-	DEL
X258	c.773_782+1del, c.772_782+2del, c.774_782+4del	3	-	DEL
X259	c.776_782+31del, c.776_782+5del	2	-	DEL
X260	c.779_782+22del, c.778_782+9del	2	-	DEL
X261	c.782+1G>A, c.782+1G>C, c.782+1G>T, c.782+2T>G, c.782+2T>C, c.782+2T>A, c.783-1G>A, c.783-1G>C, c.783-1G>T, c.783-2A>C, c.783-2A>T, c.783-2A>G, c.782+3C>G, c.783-1del, c.782_782+1del, c.783-1_787del, c.783-1_792del, c.783-19_800del, c.783-3_786del, c.782_782+1delinsTT, c.783-11_783-1del, c.783-12_786delinsAGA, c.782+164_920-44del, c.783-8_790del, c.783-2_783-1delinsTT, c.783-116_993+604del, c.783-8_783del, c.783-3_784del, c.783-15_792del, c.783-16_794del, c.782+10_895del, c.782+146_919+28del, c.782+2_782+6del, c.782+2del, c.783-4_783-3del, c.783-4G>A, c.783-5A>G, c.783-5T>C, c.783-6_783-5del, c.783-6_783-5dup, c.783-8_783-5del, c.783-8C>G	120	23	SNP, DEL, INS, DUP
X271	c.811_920-34del	1	-	DEL
X283	c.848_919+3del	1	-	DEL
X287	c.860_920-1del	1	-	DEL

X294	c.882_993+16del	1	-	DEL
X300	c.899_919+16del	1	-	DEL
X305	c.913_919+14delinsCAGCAG, c.915_919+1del	2	-	DEL
X307	c.919+1G>T, c.919+1G>A, c.919+1G>C, c.919+2T>A, c.919+2T>G, c.920-1G>A, c.920-1G>C, c.920-1G>T, c.920-2A>T, c.920-2A>C, c.920-2A>G, c.920-2del, c.920-11_920del, c.920-16_920del, c.920-29_923del, c.920-1_921del, c.920-1_920delinsAT, c.919_919+1delinsTT, c.920-1_920delinsTT, c.920-2_923del, c.920-15_933del, c.919+41_993+456del, c.920-10_926del, c.920-2_922delinsGG, c.919+2del, c.920-17_933del, c.920-30_920del, c.919+39_993+206del, c.920-16_920del, c.920-1_921del, c.920-3_920del, c.920-21_920del, c.920-4_984del, c.919+19_993+190del, c.919_919+1delinsTT, c.920-12_920-2del, c.919+37_993+200del, c.920-1_922delinsCT, c.920-1del, c.919+1del, c.919+6dup, c.919+7A>C, c.920-14_920-3del, c.920-3T>C, c.920-4C>G, c.920-5C>T, c.920-7T>G	153	37	SNP, DEL, INS, DUP
X308	c.923_993+137del	1	-	DEL
X321	c.963_993+39del	1	-	DEL
X324	c.972_993+4delinsAGGA	1	-	DEL
X325	c.974_993+4delinsAA	1	-	DEL
X327	c.979_993+27del, c.980_993+15delinsCCATCTTATC	2	-	DEL
X330	c.989_993+26del	1	-	DEL
X331	c.993G>A, c.993+1G>T, c.993+1G>A, c.993+1G>C, c.993+2T>C, c.993+2T>G, c.993+2T>A, c.991_993+4del, c.993+2_993+5del, c.991_993+5del, c.993+1del, c.993+851_1100+280del, c.993+7A>G, c.993+8G>C	104	46	SNP, DEL
X332	c.994-1G>T, c.994-1G>C, c.994-1G>A, c.994-2A>C, c.994-2A>G, c.994-2A>T, c.994-1_995inv, c.994-10_1004del, c.994-2_1009del, c.994-102_1036del, c.994-2del, c.994-3del, c.994-4A>G, c.994-4T>C, c.994-5T>C, c.994-6C>A, c.994-7del, c.994-7dup, c.994-7G>T, c.994-7T>A, c.994-8T>C	75	27	SNP, DEL, INS, DUP
X342	c.1024_1100+58del	2	-	DEL
X354	c.1061_1101-260del	1	-	DEL
X367	c.1100+1G>A, c.1101-1G>A, c.1101-2A>G, c.1101-2A>C, c.1101-2A>T, c.1101-18_1102del, c.1100+7G>A, c.1100+8A>G, c.1100+8A>T, c.1101-3C>T, c.1101-5T>C	9	7	SNP, DEL
Others	c.*98_*100+13del, c.*99T>C, c.-23A>G, c.-25C>T, c.74+7G>T, c.74+8G>A, c.918A>G, c.558T>C, c.996C>T, c.922C>T, c.72A>G, c.378C>T	-	12	SNP, DEL

Table S2. List of *TP53* target genes or those associated with *TP53* expression from ARCHS4 RNA-seq gene-gene co-expression matrix, Enrichr gene-gene co-occurrence matrix, Tagger literature gene-gene co-mentions matrix, and GeneRIF literature gene-gene co-mentions matrix.

Combined List													
AR	DDB2	KIF20A	RAVER1	ZDHHC12	PAGR1	ZNF202	ZNF561	ACTB	CTLA4	JUN	POLE	ERCC2	RCHY1
AEN	DDIAS	KIF22	RCC1	ZNF217	PAICSP4	ZNF212	ZNF562	AFP	CTNNB1	KDR	POU5F1	ESR2	RELA
ALYREF	DDX12P	KIF23	RCC2	ZNF581	PGBD4	ZNF225	ZNF565	AKT1	CXCL8	KEAP1	PPARG	FAM25C	RPL11
APEX1	DDX39A	KIF2C	REEP4	ZWINT	PIPSL	ZNF227	ZNF566	ALB	CYCS	KIT	PRKDC	FAS	RPL5
APEX2	DKC1	KIFC1	RFWD3	ALDH7A1P1	PMS2P1	ZNF230	ZNF567	ALK	DICER1	KLK3	PROM1	FHIT	S100A4
APRT	DLGAP5	KPNA2	RMI2	AMELY	PMS2P2	ZNF234	ZNF574	ANXA5	DNMT3A	KRAS	PTEN	GDF15	S100B
ARHGAP11A	DNMT1	LIG1	RNASEH2A	CA5BP1	POT1	ZNF260	ZNF576	ANXA8	DNTT	KRT19	PTGS2	GSTM1	SAMD4B
ARHGEF19	DPP3	LMNB1	RPA1	CENPBD1	PSME2P2	ZNF263	ZNF584	APAF1	E2F1	KRT20	PTPRC	GSTP1	SCO2
ASF1B	DTL	LMNB2	RPL12	CHMP4A	RBM23	ZNF264	ZNF586	ARID1A	EGF	KRT5	PXDN	GSTT1	SERPINB5
AURKB	DTYMK	LRR1	RPL18	CT47A6	RHEBP1	ZNF268	ZNF587	ASXL1	EGFR	KRT7	PXDNL	HIPK2	SERPINE1
BAX	E2F2	MCM2	RPL18A	CYCSP55	RHEBP2	ZNF274	ZNF614	ATM	EP300	LIAT1	RAD50	HNRNPK	SF3B1
BCL2L12	EFNA4	MCM3	RPL7A	DBIL5P	RHOQP1	ZNF282	ZNF616	ATRX	EPCAM	MAP2K1	RB1	HRK	SFN
BIRC5	EIF3D	MCM5	RPLP0	DEFB104B	RNPS1P1	ZNF284	ZNF619	B2M	ERBB2	MAPK3	RET	HSPA9	SIRT3
BUB1	EIF4A1	MCM6	RPS16	DEFB105A	SAA3P	ZNF286A	ZNF620	BCL2	ESR1	MCL1	RPS6KB1	IFI16	SP1
BUB1B	EIF4EBP1	MCM7	RPS19	DEFB105B	SELV	ZNF3	ZNF623	BCL2L1	FASLG	MDM2	RUNX1	ING1	THBS1
BYSL	EIF5A	MELK	RPS2	DEFB119	SEPT7P9	ZNF304	ZNF625	BCL2L11	FBXW7	MDM4	SDHC	KAT2B	TIGAR
C10orf2	ESPL1	MGME1	RPS3	DEPDC4	SMN2	ZNF317	ZNF639	BCL6	FGF2	MET	SIRT1	KAT5	TNFSF10
C17orf53	EXO1	MKI67	RPS4X	FAM149B1	SMURF2P1	ZNF318	ZNF646	BECN1	FGFR3	MGMT	SLCO6A1	KLF4	TP63
C19orf48	EZH2	MTA2	RPSA	FAM200A	SPACA5B	ZNF319	ZNF668	BRAF	FLT3	MLH1	SMAD4	KLF5	TP73
C19orf54	FAM60A	MTFR2	RRM2	GH1	STX4	ZNF324	ZNF669	BRCA1	FN1	MME	SNAI1	KMT2D	TWIST1
CAD	FAM64A	MYBL2	RUVBL1	GH2	TCEB3B	ZNF324B	ZNF672	BRCA2	FOS	MMP2	SOD2	KMT5A	UBE3A
CASP2	FAM86C1	MYC	SF3B4	GNRHR2	THAP9	ZNF330	ZNF684	BTK	FOXO3	MMP9	SOX2	LINC00385	USP7

CBX2	FANCA	NCAPD2	SHMT2	HIGD2B	TIGD6	ZNF33A	ZNF687	CASP3	GADD45A	MMUT	SRC	LINC01761	VEGFA
CCNB1	FANCD2	NCAPH	SLC16A13	HSPD1P1	TLK2P1	ZNF341	ZNF689	CASP8	GAPDH	MSH2	STAT3	MAPK1	VHL
CCNB2	FANCE	NDC80	SLC1A5	IFNA1	TP53BP1	ZNF343	ZNF691	CASP9	GFAP	MSH6	STK11	MAPK14	XPC
CCNF	FANCG	NOB1	SNRPA	INS	TP53BP2	ZNF346	ZNF696	CCK	GLB1	MTOR	SYP	MAPK8	XRCC1
CDC20	FANCI	NONO	SNRPB	KIAA0100	TTC31	ZNF354A	ZNF7	CCNA1	GPT	MYCN	TCHP	MEG3	
CDC25C	FBL	NOP2	TACC3	KIAA0391	TXNRD3NB	ZNF384	ZNF70	CCND1	GSK3B	NANOG	TERT	MIR122	
CDC45	FBXO5	NPM3	TCF19	KIAA0586	UBE2D4	ZNF394	ZNF701	CCNL2	H2AX	NCAM1	TET2	MIR125A	
CDC6	FOXM1	NRM	TCF3	KIAA0753	VDAC1P1	ZNF398	ZNF74	CD19	H3C12	NF1	TGFB1	MIR34A	
CDCA2	GATAD2A	NUP62	TGIF2	KIAA1143	VDAC1P3	ZNF408	ZNF740	CD274	H3C13	NFE2L2	TNF	MIR34B	
CDCA4	GEMIN4	NUSAP1	THOC6	KRT8P12	VDAC1P6	ZNF410	ZNF746	CD34	HDAC9	NFKB1	WT1	MIR34C	
CDCA5	GINS2	ORC1	TIMELESS	LRRC37BP1	XRCC6P2	ZNF417	ZNF747	CD38	HIF1A	NFKBIA	XIAP	MTHFR	
CDCA7	GINS4	PABPC1	TK1	MCTS2P	YY1AP1	ZNF426	ZNF749	CD4	HMOX1	NOTCH1	ABCB1	NBN	
CDCA8	GRWD1	PCNA	TNFRSF10B	METTL2A	ZBED1	ZNF444	ZNF76	CD44	HRAS	NPM1	ADAM11	NCL	
CDK2	GSG2	PFAS	TONSL	METTL2B	ZNF101	ZNF45	ZNF764	CD68	HSP90AA1	NRAS	APC	NEDD8	
CDK4	GTSE1	PFN1	TOP2A	MORC2	ZNF131	ZNF473	ZNF766	CD8A	HSP90AB1	PALB2	ATF3	NME1	
CDT1	HDAC1	PLK1	TPX2	MRPS31P4	ZNF134	ZNF48	ZNF768	CDH1	HSPA4	PARP1	ATR	NOS2	
CENPA	HJURP	POC1A	TRAIP	MTERF1	ZNF142	ZNF480	ZNF776	CDH2	IDH1	PDCD1	AURKA	NQO1	
CENPH	HMGA1	POLA2	TRIM28	OR13C8	ZNF146	ZNF490	ZNF777	CDK1	IDH2	PDGFRA	BAK1	NUMB	
CENPO	HN1L	POLD1	TROAP	OR2AG1	ZNF155	ZNF500	ZNF784	CDK6	IFNG	PECAM1	BBC3	OGG1	
CEP55	HNRNPA1	PPM1G	TTK	OR2F2	ZNF16	ZNF510	ZNF785	CDKN1A	IGF1	PGR	BID	PDCD5	
CHAF1A	HNRNPAB	PPRC1	TUBA1C	OR52J3	ZNF17	ZNF511	ZNF79	CDKN1B	IGF1R	PIK3C3	CASP6	PIN1	
CHEK2	HNRNPF	PSME3	TYMS	OR52W1	ZNF174	ZNF526	ZNF799	CDKN2A	IL10	PIK3CA	CCNE1	PML	
CHST14	HNRNPUL1	PTBP1	UBE2C	OR56A5	ZNF18	ZNF527	ZNF805	CDKN2B	IL1A	PIK3CB	COP1	PPM1D	
CKS1B	IMPDH2	PTTG1	UBE2I	OR5D18	ZNF180	ZNF543	ZNF830	CDKN3	IL1B	PIK3CD	CREBBP	PPP1R13L	
CKS2	IPO4	RACK1	UHRF1	OR5I1	ZNF197	ZNF548	ZNF839	CEACAM5	IL2	PIK3CG	CYP1A1	PPP2R2A	
DAXX	KIF11	RAD51	WRAP53	OR8B4	ZNF2	ZNF551	ZSCAN32	CHEK1	IL6	PMAIP1	CYP2E1	PRKAA1	
DCTPP1	KIF18B	RAD54L	XRCC3	PAAF1	ZNF200	ZNF557	ABL1	CREB1	JAK2	PMS2	ERCC1	PTK2	
